# CoTAR: Topology and Atomic State Reconstruction in Condensed Phases

**DOI:** 10.1021/acs.jcim.6c02184

**Published:** 2026-09-18

**Authors:** Hodaka Mori, Yu Miyazaki, Takechika Kikkawa

**Affiliations:** 508827 Preferred Networks, Inc., Tokyo 100-0004, Japan

## Abstract

Universal machine learning interatomic potentials (uMLIPs) enable performing condensed-phase molecular dynamics (MD) simulations with accuracy approaching that of first-principles; however, their lack of explicit molecular topology limits bond-aware analysis and reconnection to classical force fields. This study presents CoTAR, a hybrid graph neural network (GNN)–hidden Markov model (HMM) framework that reconstructs bond connectivity and order, formal charges, and unpaired electrons from atomic species and coordinates by combining learned local environments, chemical constraints, and temporal smoothing. After joint fine-tuning with 20 labeled snapshots per benchmark system, CoTAR achieved category-macro bond and conditional bond-order F1 scores of 0.992 and 0.998 and chemically valid snapshots of 82.1% across 128 uMLIP systems. Zero-shot tests were successful for all tested nonionic systems but revealed persistent formal-charge errors in ionic mixtures. For successfully reconnected systems, the reconstructed-topology simulation results showed close agreement with the reference-topology results for the density, element-resolved diffusion, and intermolecular radial distribution functions. These results establish CoTAR as a practical route from topology-free uMLIP trajectories to chemically complete molecular representations within the present scope of nonreactive, topology-preserving systems.

## Graphical abstract

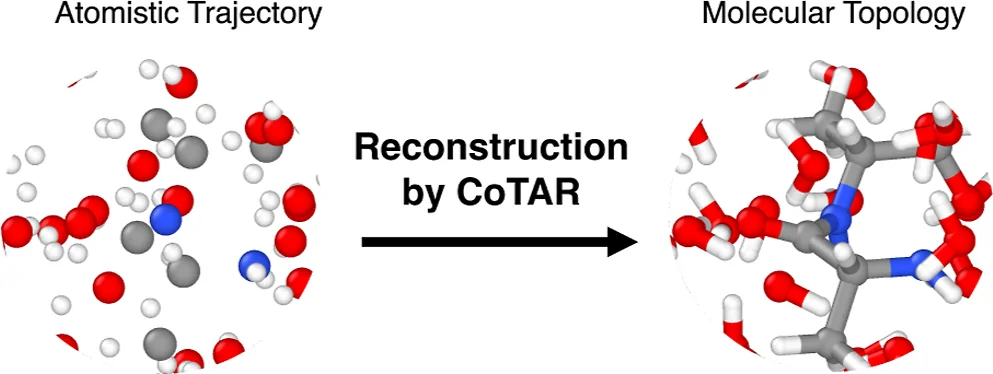

## Introduction

Universal machine learning interatomic potentials (uMLIPs) have enabled carrying out condensed-phase molecular dynamics (MD) simulations with near-first-principles accuracy at practical and feasible computational costs.^1–5^ This capability is important for liquids, solutions, polymers, and interfaces, where quantum chemical treatments are often computationally expensive and classical force fields may lack transferability.^6,7^

In contrast to classical force fields, uMLIP-based simulations generally do not require explicit molecular topology.^1–5^ Although this flexibility is advantageous, it complicates analyses depending on bonding information, including molecule identification, reaction tracking, and functional-group analysis. Furthermore, it complicates reconnection of selected uMLIP configurations or pathways to classical force fields for large-scale or long-time-scale simulations.

Distance heuristics are widely used to infer covalent bonds from coordinates;^8–10^ however, condensed phases contain numerous short intermolecular contacts, including hydrogen bonds, solvation-shell contacts, ion pairs, and dense packing contacts. Consequently, these methods can generate false intermolecular bonds and do not assign bond orders. Constraint-based bond-order assignment methods can recover bond orders by enforcing valence and charge consistencies on a candidate connectivity graph.^11^ However, in dense periodic systems, numerous short intermolecular contacts render a candidate graph larger and more ambiguous, which can increase search cost and lead to practical failures.

Graph neural networks (GNNs) and temporal smoothing schemes such as hidden Markov models (HMMs) have been employed to infer bonds, but most of such reported approaches focus on isolated molecules or dilute systems.^12–15^ Condensed-phase trajectories remain challenging because close intermolecular contacts must be distinguished from covalent bonds under periodic boundary conditions (PBCs) while preserving the consistency of formal charges and unpaired electrons.

This study presents CoTAR (Coordinates-to-Topology and Atom-State Reconstruction), a hybrid GNN–HMM framework, for reconstructing molecular topology and atom states from condensed-phase trajectories under PBCs. CoTAR predicts pair states and atom states from atomic species, coordinates, and optional system net charge and combines a van der Waals (vdW) prior with valence and charge constraints to suppress spurious intermolecular bonds and chemically inconsistent assignments. For uMLIP trajectories, we apply fine-tuning in a pooled design: a single pretrained model is jointly updated on 2560 labeled snapshots from 128 benchmark systems (20 per system) and evaluated on 180 disjoint held-out snapshots per system to address the distribution shift from classical-MD trajectories. Separately, we evaluate zero-shot transfer on six complete external systems excluded from all training and target-system tuning. Downstream utility is assessed by reconnecting the reconstructed topologies to classical MD and comparing the density, diffusion coefficients, and radial distribution functions with those obtained from reference-topology simulations. By integrating learned local geometric context, explicit chemical constraints, atom-state prediction, and temporal smoothing, CoTAR provides a practical framework for converting topology-free condensed-phase trajectories into chemically complete representations for bond-aware analysis and force-field reconnection. Note that the present study focuses on nonreactive, topology-preserving systems.

## Methods

### Overview of the CoTAR Framework

The objective is to infer bond topology and atom states from atomic species *Z*_*i*_ and 3D coordinates **r**_*i*_ and optionally from the total charge *Q*_total_, while satisfying valence and charge constraints. In condensed phases, purely distance-based decisions are prone to false positives because of the dense packing and thermal fluctuations. Bond order, formal charge, and unpaired electron count are coupled through electron count and valence rules; hence, separate predictions readily yield chemically inconsistent assignments. CoTAR therefore combines (i) message-passing neural network (MPNN)-based pair-state prediction on a proximity graph, (ii) joint prediction of formal charges *q*_*i*_ and unpaired electrons *u*_*i*_, (iii) a vdW prior and chemical constraints, and (iv) HMM-based temporal refinement for trajectories.^16,17^ Figure 1 summarizes the end-to-end data flow.

**Figure 1:**
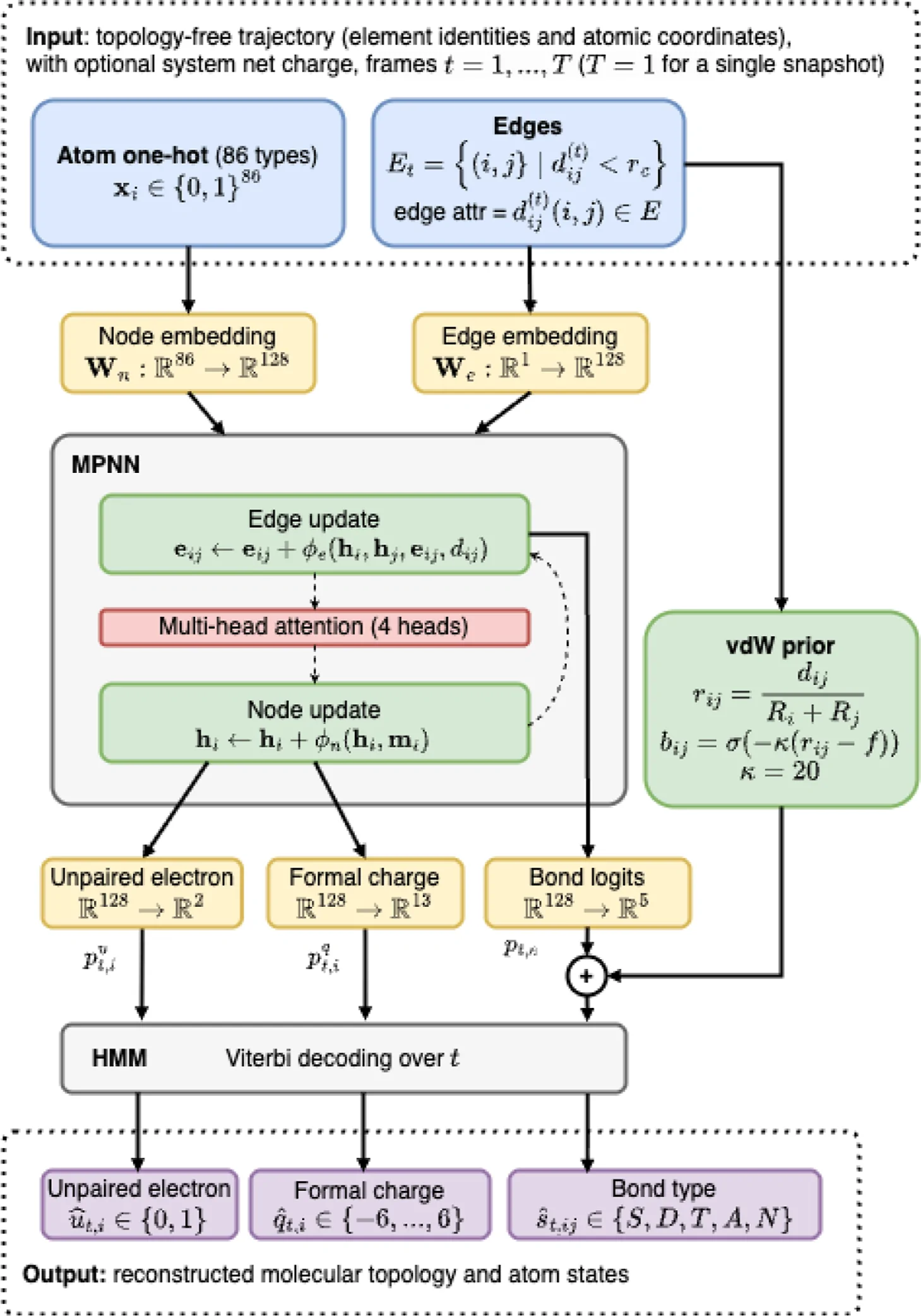
Overview and data flow of CoTAR. Inputs are trajectory frames specified by elemental identities and atomic coordinates, together with optional system net charge. A proximity graph is constructed for each frame; an MPNN jointly predicts pair and atom states; a vdW prior biases covalent versus noncovalent assignments; and HMM smoothing enforces temporal consistency. Decoded bond types (including the nonbonded state), formal charges, and unpaired-electron assignments jointly define the reconstructed molecular topology and atom states. Chemical-constraint losses encourage consistent predictions during training.

The input graph G=(V,E) is constructed by treating atoms as nodes and defining proximity pairs within a distance cutoff of *r*_c_ = 3.0 Å. To distinguish a chemical bond itself from the proximity relation handled by the GNN, we first define an undirected set of proximity pairs P and then introduce a directed edge set E for computation.

1 V={1,...,N}

2 $$P=\left\{\left\{i,j\right\}|1\leq i<j\leq N,d_{ij}<r_{c}\right\}$$

3 E={(i,j)|i≠j,⁡{i,j}∈P}

4 $$d_{ij}=∥r_{i}-r_{j}∥$$

Equations 1–4 define the atom set, undirected proximity pairs, directed computational edges, and pair distances used to construct the input graph. Under PBCs, *d*_*ij*_ is defined by the minimum-image distance. We denote the proximity neighborhood of atom *i* by N(i)={j|(i,j)∈E} . Pair labels are defined for undirected atom pairs and copied to both directed edges. At the inference time, the outputs for (*i*, *j*) and (*j*, *i*) are averaged to recover a symmetric undirected prediction.

Node features comprise only the element one-hot vector **x**_*i*_ ∈ {0,1}^86^, in which 86 channels correspond to element types with atomic numbers in the range of *Z* = 1, ..., 86 (H through Rn). Edge features comprise only the interatomic distance $d_{ij}\in R$ . This minimal representation preserves translational and rotational invariance and applies uniformly to molecules, condensed phases, and periodic systems.

Accordingly, the local environment of atom *i* is represented by the identities and minimum-image distances of atoms within the 3.0 Å distance cutoff. Repeated message passing allows information to propagate beyond a single first-neighbor shell through the proximity graph. This distance cutoff value of 3.0 Å is a CoTAR graph-construction hyperparameter and is independent of the internal neighbor cutoff used by uMLIP, which generated the input trajectory. CoTAR uses elemental identity but does not require a conventional force-field atom type as input. Such atom types are generally unavailable before topology reconstruction and will introduce a circular dependence because they are assigned from the recovered bonding environment. Environment-dependent chemical character is instead learned from the local graph and regularized by the joint bond-order, formal-charge, unpaired-electron, valence, and total-charge objectives.

The state space for proximity pairs is defined as

5 $$C_{cov}=\left\{Single,Double,Triple,Aromatic\right\}$$

6 $$C=\left\{\phi \right\}\cup C_{cov},,\left|C\right|=5$$

Equations 5 and 6 define the covalent and complete pair-state spaces, respectively. Here, ϕ denotes a noncovalent state in which a pair is spatially close but does not form a covalent bond. Treating aromatic moieties as an explicit class reflects the nonuniqueness of Kekulé representations for aromatic rings. Subsequently, we refer to these as pair states.

### GNN-Based Pair-State Prediction

Covalent bonding is not determined by pair distance alone but depends on many-body context, such as coordination number, local density, neighboring element composition, and aromaticity. CoTAR therefore employs message passing to repeatedly aggregate local environmental information and infer pair states conditioned on the surrounding structures. Condensed phases often contain numerous neighbors within the distance cutoff; hence, multistep propagation is important for distinguishing close contacts from true covalent bonds. We used *L* = 16 message-passing layers to ensure a sufficiently large receptive field. Residual connections together with LayerNorm stabilize deep training. Neighbor contributions are weighted via 4-head attention.

For a hidden dimension *D* = 128, the initial node and edge embeddings are defined by the linear maps in eqs 7–10

7 $$h_{i}^{\left(0\right)}=W_{n}x_{i}$$

8 $$e_{ij}^{\left(0\right)}=W_{e}d_{ij}$$

9 $$W_{n}:R^{86}\to R^{D}$$

10 $$W_{e}:R\to R^{D}$$

At each layer *l* = 0, ..., *L* – 1, the edge feature is first updated according to eq 11

11 $$e_{ij}^{\left(l+1\right)}=e_{ij}^{\left(l\right)}+\phi_{e}\left([h_{i}^{\left(l\right)},h_{j}^{\left(l\right)},e_{ij}^{\left(l\right)},d_{ij}]\right)$$

The distance *d*_*ij*_ is provided explicitly at every layer to preserve geometric scale information through the network depth. Messages are then aggregated via 4-head attention, as specified by eqs 12 and 13

12 $$\alpha_{ij}^{\left(l,h\right)}={softmax}_{j\in N\left(i\right)}\phi_{att}^{\left(h\right)}\left(e_{ij}^{\left(l+1\right)}\right)$$

13 $$m_{i}^{\left(l\right)}={concat}_{h=1}^{4}\sum_{j\in N\left(i\right)}\alpha_{ij}^{\left(l,h\right)}W_{m}^{\left(h\right)}e_{ij}^{\left(l+1\right)}$$

Finally, the node feature is updated using eq 14

14 $$h_{i}^{\left(l+1\right)}=h_{i}^{\left(l\right)}+\phi_{n}\left([h_{i}^{\left(l\right)},m_{i}^{\left(l\right)}]\right)$$

Both ϕ_*e*_ and ϕ_*n*_ are 2-layer MLPs with LayerNorm and SiLU.

The final edge representation is converted to pair-state logits and probabilities using eqs 15–17

15 $$z_{ij}=W_{pair}e_{ij}^{\left(L\right)}+b_{pair}$$

16 $${\mathrm{p̂}}_{ij}=softmax\left({\mathrm{z̃}}_{ij}\right)$$

17 $${\mathrm{p̂}}_{ij}\left(c\right)={\left({\mathrm{p̂}}_{ij}\right)}_{c},,c\in C$$

Here, ${\mathrm{p̂}}_{ij}\left(c\right)$ is the predicted probability that the proximity pair {*i*, *j*} belongs to state *c*.

Simultaneously, the final node representation predicts formal charges *q*_*i*_ ∈ {−6, ..., 6} and unpaired electrons *u*_*i*_ ∈ {0, 1} according to eq 18

18 $$z_{i}^{q}=\phi_{q}\left(h_{i}^{\left(L\right)}\right),,z_{i}^{u}=\phi_{u}\left(h_{i}^{\left(L\right)}\right)$$

As pair states and (*q*_*i*_, *u*_*i*_) are coupled through valence constraints, the joint atom-state prediction acts as a chemical regularizer that suppresses false bonds accompanied by valence violations.

A physically motivated vdW prior is further incorporated into the logits.^18^ Here, *R*_*i*_ denotes the element-specific van der Waals radius of atom *i*. The values used in this study were taken from the Mendeleev database.^18^ The distance ratio is defined as *r*_*ij*_ = *d*_*ij*_/(*R*_*i*_ + *R*_*j*_), and we map it into *b*_*ij*_ ∈ (0, 1) using the sigmoid in eq 19

19 $$b_{ij}=\sigma \left(-\kappa \left(r_{ij}-f\right)\right),,\kappa =20$$

The threshold-like parameter *f* (initialized to 0.6) and scale γ (initialized to 100) are learnable. This prior acts primarily on separation between the covalent states and the noncovalent state ϕ, while bond-order discrimination is left to the MPNN representation, as specified by eqs 20 and 21

20 $${\mathrm{z̃}}_{ij}\left(c\right)=z_{ij}\left(c\right)+\gamma b_{ij},,c\in C_{cov}$$

21 $${\mathrm{z̃}}_{ij}\left(\phi \right)=z_{ij}\left(\phi \right)+\gamma \left(1-b_{ij}\right)$$

### HMM-Based Temporal Refinement

In MD simulations, coordinates fluctuate continuously, while pair states usually do not change abruptly between adjacent frames. Frame-independent predictions therefore tend to contain high-frequency noise, including spike-like state flips in borderline situations. To suppress such noise while preserving meaningful state changes, we apply an HMM to the sequence of the CoTAR output probabilities and infer the most likely hidden-state trajectory using the Viterbi algorithm.^19^ No smoothing is applied for a single frame (*T* = 1).

For time *t* and proximity pair *e*, let the observation be ${\mathrm{p̂}}_{t,e}\in {\left[0,1\right]}^{\left|C\right|}$ and the hidden state be $z_{t,e}\in \left\{1,...,\left|C\right|\right\}$ . Transitions are controlled by a self-transition probability *p*_self_ defined in eqs 22–24

22 $$A_{kk}=p_{self}$$

23 $$A_{kl}=\frac{1-p_{self}}{\left|C\right|-1},\left(l\neq k\right)$$

24 $$\pi_{k}=\frac{1}{\left|C\right|}$$

The emission log-probability is taken directly from the CoTAR probability, with numerical stabilization given in eq 25

25 $$\log P\left(o_{t,e}|z_{t,e}=k\right)=\log\left({\mathrm{p̂}}_{t,e}\left(k\right)+\varepsilon \right),,\varepsilon =10^{-10}$$

The most likely sequence is given by eq 26

26 $${ẑ}_{1:T,e}=\arg\max_{z_{1:T,e}}\left[\pi_{z_{1,e}}\prod_{t=1}^{T}P\left(o_{t,e}|z_{t,e}\right)\prod_{t=2}^{T}A_{z_{t-1,e},z_{t,e}}\right]$$

To reduce the computational cost, we fix the set of pairs to be smoothed as a union of proximity pairs over representative frames rather than over the whole trajectory. If *N*_hmm_ representative frames are selected, their number is given by eq 27, as follows

27 $$N_{rep}=\min\left(T,N_{hmm}\right)$$

The representative frames are chosen uniformly over the whole trajectory. If a pair $e\in P_{hmm}$ no longer satisfies the proximity condition at time *t*, its observation is imputed as a one-hot vector for the noncovalent state ϕ. The same HMM procedure is applied to formal charges (13 states) and unpaired electrons (2 states). This study uses *p*_self_ = 0.99 and *N*_hmm_ = 20. Here, *N*_hmm_ controls only the number of representative frames used to define $P_{hmm}$ ; note that it is not a temporal block length. Viterbi decoding is applied to the complete input sequence for each system.

### Loss Functions and Chemical Constraints

Pair-state classification is local and, by itself, does not guarantee global chemical constraints, such as total-charge conservation and valence consistency. We therefore add atom-level terms that penalize violations of the electron-count consistency. The total loss is given by eq 28

28 $$L=L_{pair}+L_{atom}$$

For each annotated snapshot (*c*, *t*), we define the valid directed-edge and atom sets in eqs 29 and 30, respectively, as follows

29 $$E_{valid}^{\left(c,t\right)}=\left\{\left(i,j\right)|\left\{i,j\right\}\in P_{c}^{\left(t\right)}\right\}$$

30 $$V_{valid}^{\left(c,t\right)}=V_{c}$$

During implementation, the loss is averaged over the union of these sets in a minibatch.

Thus, the pair-state classification loss is the average cross-entropy over the valid directed edges in eq 31

31 $$L_{pair}=\frac{1}{\left|E_{valid}\right|}\sum_{\left(i,j\right)\in E_{valid}}CE\left(l_{ij}^{*},{\mathrm{p̂}}_{ij}\right)$$

Here, $l_{ij}^{*}\in C$ is the reference state assigned to the corresponding undirected proximity pair and copied to both directed edges. We use standard cross-entropy rather than focal loss or explicit class weighting because of the candidate restriction imposed by the distance cutoff, the physical bias from the vdW prior, and effective imbalance mitigation by the atom-level terms.

The atom-level loss is defined in eq 32 as a linear combination of multiple constraints, regularizers, and auxiliary supervision terms, as follows

32 $$L_{atom}=\sum_{\tau \in T}\lambda_{\tau }L_{\tau }$$

where T={cons,Q,u,|q|,qCE,uCE} . The constraint terms are evaluated in expectation using the atom-state expectations in eq 33 to avoid nondifferentiability

33 $${\mathrm{q̂}}_{i}=\sum_{c=-6}^{6}cP\left(q_{i}=c\right),,{û}_{i}=\sum_{u\in \left\{0,1\right\}}uP\left(u_{i}=u\right)$$

The formal-charge label space is fixed to *c* ∈ {−6, ..., 6} for covering the range of charge states that may arise for the element types considered in this study (*Z* = 1, ..., 86).

The predicted valence induced by pair states is defined as the sum of expected bond orders in eqs 34 and 35

34 $$v_{i}^{pred}=\sum_{j\in N\left(i\right)}{\mathrm{b̅}}_{ij}$$

35 $${\mathrm{b̅}}_{ij}=1\cdot {\mathrm{p̂}}_{ij}\left(Single\right)+2\cdot {\mathrm{p̂}}_{ij}\left(Double\right)+3\cdot {\mathrm{p̂}}_{ij}\left(Triple\right)+1.5\cdot {\mathrm{p̂}}_{ij}\left(Aromatic\right)$$

The noncovalent state ϕ does not contribute to ${\mathrm{b̅}}_{ij}$ . Valence consistency is then enforced through eqs 36 and 37

36 $$v_{i}^{eff}=v_{i}^{std}-{\mathrm{q̂}}_{i}-{û}_{i}$$

37 $$L_{cons}=\frac{1}{\left|V_{valid}\right|}\sum_{i\in V_{valid}}{\left(v_{i}^{pred}-v_{i}^{eff}\right)}^{2}$$

Here, *v*_*i*_ denotes the default standard valence of element *Z*_*i*_. Representative values for the principal main-group elements included in this study are H = 1, C = 4, N = 3, O = 2, F/Cl/Br/I = 1, P = 3, and S = 2.

Total-charge consistency is imposed as the batch-level term in eq 38

38 $$L_{Q}=\frac{1}{B}\sum_{b=1}^{B}{\left(\sum_{i}{\mathrm{q̂}}_{b,i}-Q_{b}\right)}^{2}$$

where *Q*_b_ = 0 if the total charge is unspecified.

To reflect the predominance of closed-shell states in condensed phases, we impose weak penalties on the radical character and excessive charge given in eqs 39 and 40

39 $$L_{u}=\frac{1}{\left|V_{valid}\right|}\sum_{i\in V_{valid}}{û}_{i}$$

40 $$L_{\left|q\right|}=\frac{1}{\left|V_{valid}\right|}\sum_{i\in V_{valid}}\left|{\mathrm{q̂}}_{i}\right|$$

When ground-truth formal charges and unpaired electrons are available, the cross-entropy terms in eqs 41 and 42 cannot be excluded

41 $$L_{qCE}=\frac{1}{\left|V_{valid}\right|}\sum_{i\in V_{valid}}CE\left(z_{i}^{q},q_{i}^{gt}\right)$$

42 $$L_{uCE}=\frac{1}{\left|V_{valid}\right|}\sum_{i\in V_{valid}}CE\left(z_{i}^{u},u_{i}^{gt}\right)$$

For formal-charge classification, softmax is restricted to chemically allowed charge states for each element and an unpaired-electron state, thereby preventing probability mass from being assigned to impossible states. The weights are fixed throughout training at (cons, *Q*, *u*, |*q*|, *q*CE, *u*CE) = (0.5, 0.1, 0.1, 0.1, 1.0, 1.0).

### Data Curation and Datasets

We use classical-MD trajectories from all 128 systems for pretraining and uMLIP trajectories from the same systems for fine-tuning and evaluation. The systems are nonreactive and topology-preserving under PBCs, contain approximately 1000–3000 atoms, and are simulated at approximately 300 K and 1 atm. The uMLIP dataset contains approximately 25,000 frames.

The systems are grouped into 109 PURELIQUID, 5 SOLUTION, 9 ION, and 5 RADICAL systems. The ION subset comprises NaCl, CaCl_2_, and NaOH solutions at three concentrations, and the RADICAL subset comprises five open-shell species. Because 109 of the 128 benchmark systems are PURELIQUID, a fully pooled summary is dominated by that category. We therefore use an unweighted macroaverage over the PURELIQUID, SOLUTION, ION, and RADICAL subsets as the primary aggregate and report each category separately. This reporting choice does not remove the underlying imbalance; the limited ionic and radical diversity remains a limitation. The complete inventory is given in Supporting Information.

Six additional complete condensed-phase systems spanning four categories were used for zero-shot evaluation. Each external system is excluded in its entirety from classical MD pretraining, uMLIP fine-tuning, model selection, and target-system parameter tuning. All model weights and inference settings were fixed before evaluating these systems. Elements and some constituent species or local motifs can overlap with the benchmark inventory; therefore, the experiment measures transfer to unseen systems rather than performing full chemistry-disjoint extrapolation. The external set supplies additional challenging ionic and radical examples but is not presented as a balanced replacement for the 128-system benchmark. System compositions are listed in Table S1.

### Reference Annotation and Data Splits

For each system *c*, the reference topology is defined by eq 43

43 $$T_{c}^{ref}=\left(V_{c},B_{c},\beta_{c},q_{c},u_{c}\right)$$

where $B_{c}$ is a set of undirected atom pairs that are covalently bonded, β_c_ assigns their covalent states (single, double, triple, and aromatic), and *q*_c_ and *u*_c_ denote atom-wise formal charges and unpaired electrons, respectively. The reference topology for each system is extracted once from the RDKit molecule generated from the input SMILES and shared across all frames.^11,20^ Bond information is read from bond objects, while formal charges and unpaired-electron counts are read from atom attributes. The same procedure is used for both datasets.

For each snapshot *t*, an undirected set of proximity pairs is constructed according to eq 44

44 $$P_{c}^{\left(t\right)}=\left\{\left\{i,j\right\}|i<j,d_{ij}^{\left(t\right)}<r_{c}\right\}$$

Each pair $\left\{i,j\right\}\in P_{c}^{\left(t\right)}$ is then assigned a reference state in eq 45

45 $$l_{ij}^{*\left(t\right)}=\{\begin{array}{ll} \beta_{c}\left(i,j\right), & \left\{i,j\right\}\in B_{c},\\ \phi , & \left\{i,j\right\}\in P_{c}^{\left(t\right)}\backslash B_{c} \end{array}$$

Thus, a proximity pair receives a covalent state if it belongs to the reference topology; otherwise, it receives a noncovalent state ϕ. Atom-wise labels are defined by eq 46

46 $$q_{i}^{*\left(t\right)}=q_{c}\left(i\right),,u_{i}^{*\left(t\right)}=u_{c}\left(i\right)$$

and are inherited across frames through conserved atom indexing. During training, each undirected pair-state label is duplicated onto the two directed edges (*i*, *j*) and (*j*, *i*) handled by the GNN.

At evaluation time, the proximity-pair set is generated with the same distance cutoff of *r*_c_ = 3.0 Å. No special handling is introduced to add back reference bonds outside this cutoff.

For the classical MD benchmark, training and validation are split at the frame level within each system (90%/10%). This design emphasizes robustness to thermal fluctuations and local environment variations within the represented systems rather than extrapolation to unseen systems. All 128 benchmark systems are included in the classical-MD evaluation results reported in Table 1A.

**Table 1:** Category-Macro and Category-Resolved Results for the Classical-MD (A) and uMLIP (B) Benchmarks^a^

category macro		by category			
method	macro avg	pure liquid	solution	ion	radical
(A) classical-MD evaluation set (128 systems)					
conditional bond-order F1 (↑)					
RDKit	0.278	0.413	0.262	0.000	0.436
CoTAR	0.999	0.997	1.000	1.000	0.998
valid snapshots (%) (↑)					
vdW	46.3	90.7	11.7	0.0	83.0
vdW + HMM	52.4	94.7	26.6	4.9	83.3
RDKit	21.1	31.1	20.0	0.0	33.3
CoTAR	94.0	100.0	100.0	99.4	76.5
avg. problems/snapshot (↓)					
vdW	12.9	1.16	19.7	27.5	3.00
vdW + HMM	5.92	0.769	6.90	13.0	3.00
RDKit	1832	1, 980	2355	2070	923
CoTAR	0.170	0	0	0.006	0.675
(B) uMLIP evaluation benchmark (128 systems)					
bond F1 (↑)					
vdW	0.9814	0.9990	0.9855	0.9418	0.9992
vdW + HMM	0.9815	0.9990	0.9856	0.9422	0.9993
RDKit	0.6561	0.9416	0.9274	0.0000	0.7555
CoTAR	0.9924	0.9999	0.9999	0.9700	1.0000
conditional bond-order F1 (↑)					
RDKit	0.6383	0.8822	1.0000	0.0000	0.6711
CoTAR	0.9984	0.9955	1.0000	1.0000	0.9981
formal-charge F1 (↑)					
RDKit	0.6513	0.9364	0.9189	0.0000	0.7498
CoTAR	0.9969	0.9946	1.0000	0.9931	1.0000
unpaired-electron F1 (↑)					
RDKit	0.6520	0.9396	0.9189	0.0000	0.7496
CoTAR	0.9996	1.0000	1.0000	1.0000	0.9983
valid snapshots (%) (↑)					
vdW	36.8	70.8	0.1	0.0	76.3
vdW + HMM	37.6	71.8	0.1	0.0	78.7
RDKit	58.2	88.6	84.0	0.0	60.0
CoTAR	82.1	91.9	85.7	61.0	89.8
avg. problems/snapshot (↓)					
vdW	88.12	7.82	131.15	206.53	6.98
vdW + HMM	87.49	7.73	130.58	204.74	6.90
RDKit	3, 414.88	994.47	1, 203.76	7, 916.11	3, 545.20
CoTAR	0.82	0.28	0.31	2.49	0.20

a Conditional bond-order F1 includes pairs covalent in both reference and prediction; vdW baselines predict only connectivity.

For the main uMLIP transfer evaluation, a fixed manifest defines exactly 200 frames from each system before any method is executed. Twenty labeled frames from each system are pooled into a 2560-frame training set and used to jointly fine-tune a single pretrained CoTAR model; furthermore, the remaining 180 frames form a common held-out evaluation set. CoTAR, vdW, vdW + HMM, and RDKit are evaluated on these identical 180 frames. For HMM-based methods, the held-out frames are supplied in chronological order and decoded as a complete held-out sequence; the RDKit procedure is applied independently to each held-out snapshot under the 5 s per-snapshot time limit defined below. No frame is selected or excluded according to a method’s prediction, completion status, failure, or timeout. Failed and timed-out assignments remain in the relevant denominators and are scored as unknown.

For each external system, 200 frames are uniformly subsampled from the 5001-frame production trajectory and processed chronologically. Except that target-system fine-tuning is omitted and all 200 frames are evaluated, all other data generation and evaluation conditions match the main uMLIP benchmark. Moreover, the model and all settings are fixed by using only the 128 benchmark systems before evaluation.

### Training Protocol

Training is performed in two stages to transfer a general condensed-phase representation from classical-MD data to uMLIP trajectories. We use AdamW with a batch size of 8 throughout.^21^ The model is first pretrained on the classical-MD dataset for 1 epoch at a learning rate of 2 × 10^–4^. For main uMLIP transfer evaluation, a single pretrained model is fine-tuned jointly on the pooled set of 2560 labeled frames, comprising exactly 20 frames from each of the 128 benchmark systems, for 10 epochs at a learning rate of 1 × 10^–4^, with all other hyperparameters kept unchanged.

For the controlled ablation study results in Table 2, we use a separate adaptation protocol to compare model components under matched conditions. For each compound, 500 snapshots are sampled from the uMLIP trajectories; 450 frames are used for fine-tuning, and 50 frames are used for evaluation. Each ablation variant was trained for 5 epochs from the same pretrained initialization. This protocol differs from the main transfer evaluation; Table 2 therefore compares only the component variants and is not directly comparable to Table 1B.

**Table 2:** Ablation of CoTAR Using 450 Fine-Tuning and 50 Evaluation Frames per Compound^a^

comp			metrics				
vdW	chem	HMM	BO ↑	FC ↑	UE ↑	valid (%) ↑	avg. prob. ↓
√	√	√	0.9970	0.9966	0.9999	98.9	0.02
√	√		0.9945	0.9911	0.9999	97.7	0.04
	√		0.9970	0.9972	1.0000	97.4	0.24
√			0.9972	0.9899	0.9998	95.0	6.23
			0.9971	0.9899	0.9998	95.3	3.07

a Checkmarks denote enabled components; BO, FC, and UE are conditional bond-order, formal-charge, and unpaired-electron F1, respectively. Bond F1 is 1.000 for all variants.

### Evaluation Metrics and Baselines

We evaluate topology reconstruction from three perspectives: local classification accuracy, practical completion, and snapshot-level chemical validity. Although CoTAR predicts pair states in the set $C=\left\{\phi \right\}\cup C_{cov}$ , the main figures also report the derived metrics bond and bond order for readability.

#### Local Metrics

For each proximity pair, we define the binary reference label in eq 47

47 $$a_{ij}^{*\left(t\right)}=1\left[l_{ij}^{*\left(t\right)}\neq \phi \right]$$

and define the predicted pair state and binary bond label using eq 48

48 $${\mathrm{l̂}}_{ij}^{\left(t\right)}=\arg\max_{c\in C}{\mathrm{p̂}}_{ij}^{\left(t\right)}\left(c\right),,{â}_{ij}^{\left(t\right)}=1\left[{\mathrm{l̂}}_{ij}^{\left(t\right)}\neq \phi \right]$$

Bond is the F1 score for bonded versus nonbonded classification over undirected proximity pairs $\left\{i,j\right\}\in P_{c}^{\left(t\right)}$ . Bond order is the support-weighted F1 score over the four covalent classes

$$C_{cov}=\left\{Single,Double,Triple,Aromatic\right\}$$

restricted to the pair set defined in eq 49

49 $$P_{BO,c}^{\left(t\right)}=\left\{\left\{i,j\right\}\in P_{c}^{\left(t\right)}|l_{ij}^{*\left(t\right)}\in C_{cov},{\mathrm{l̂}}_{ij}^{\left(t\right)}\in C_{cov}\right\}$$

Accordingly, when a true covalent bond is predicted as ϕ, it contributes a false negative to the bond metric but is excluded from bond order evaluation. We therefore refer to this quantity as conditional bond-order F1: it measures bond-order discrimination conditional on both the reference and prediction being covalent and must be interpreted together with the bond and valid metrics. Additionally, formal charge (13 classes) and unpaired electron (2 classes) are evaluated using the weighted F1 scores.

For each multiclass task, weighted F1 is the support-weighted mean of class-wise F1 scores; classes with zero reference support have zero weight. Outputs unresolved because of failure or timeout are scored as incorrect rather than excluded. Method–metric combinations not defined for a baseline are omitted from the comparison table.

#### Snapshot-Level Metrics

For high-throughput use, success is defined as the fraction of frames for which inference completes within the prescribed time limit and a topology is returned. To assess downstream usability, we introduce two per-snapshot metrics based on RDKit chemical-sanity diagnostics. Let ${\mathrm{M̂}}_{i}$ denotes a molecular object reconstructed from the prediction, and let $n_{chem}\left({\mathrm{M̂}}_{i}\right)$ be the number of chemically problematic features reported by RDKit. If the bond order, formal charge, or unpaired electrons remain undetermined because of failure or timeout, these unknown assignments are counted as downstream failures and added to the total problem count in eq 50.

50 $$n_{cp}^{\left(i\right)}=n_{chem}\left({\mathrm{M̂}}_{i}\right)+\sum_{t\in \left\{BO,FC,UE\right\}}n_{unk,t}^{\left(i\right)}$$

We then define valid in eq 51

51 $$Valid=\frac{1}{N}\sum_{i=1}^{N}1\left[n_{cp}^{\left(i\right)}=0\right]$$

as the fraction of frames with neither RDKit-detected chemical problems nor unknown assignments. The average problem count is defined in eq 52

52 $$Ave.Problems=\frac{1}{N}\sum_{i=1}^{N}n_{cp}^{\left(i\right)}$$

as the average number of diagnostic problems and unknown assignments per snapshot. We emphasize valid because downstream use requires a topology that is chemically complete and internally consistent, not just locally accurate on pairwise labels.

#### Aggregation

For Table 1, classification confusion matrices are pooled over all systems within each category before the corresponding F1 score is calculated. Valid and Ave. problems are aggregated over snapshots within each category. Because the benchmark contains 109 PURELIQUID, 5 SOLUTION, 9 ION, and 5 RADICAL systems, the primary “Macro avg.” is the unweighted arithmetic mean of the four category-specific values; therefore, each chemical category contributes equally despite the unequal system counts. Category-resolved values are reported alongside the macro-average to determine performance on smaller subsets. Aggregation details and classical-MD sensitivity estimates for system-macro and fully pooled summaries are reported in Supporting Information.

#### Baselines

We compare CoTAR with (i) a vdW-radius heuristic, with and without HMM smoothing, and (ii) a valence-rule-based baseline denoted as RDKit.

For the vdW baseline, a pair is classified as covalently bonded according to eq 53

53 $$P_{vdW}^{\left(t\right)}=\left\{\left\{i,j\right\}|d_{ij}^{\left(t\right)}<f\left(R_{i}^{vdW}+R_{j}^{vdW}\right)\right\}$$

where *R*_*i*_^vdW^ denotes the element-specific van der Waals radius (in Å) of atom *i* taken from the Mendeleev database.^18^ For consistency with the vdW prior in eq 19, the scaling parameter is fixed to *f* = 0.6 in this baseline. The distance baseline predicts only connectivity. To construct an RDKit molecule for snapshot-level chemical-sanity diagnostics, all predicted bonds are instantiated as single and atoms are assigned neutral, closed-shell default states. Bond order, formal charge, and unpaired electron F1 scores are therefore not reported for this baseline; the resulting valid value measures the chemical sanity of this default reconstruction and should not be interpreted as evidence that atom states are predicted. For vdW + HMM, HMM smoothing is applied to the binary bonded/nonbonded state over the same chronologically ordered frame sequence used for CoTAR. All distance calculations under PBCs use the minimum-image convention.

For the RDKit baseline, the built-in DetermineBonds procedure is not directly applicable because it does not support PBCs. We therefore initially construct a PBC-aware candidate-neighbor set from the minimum-image distances and then assign bond orders using an xyz2 mol-style valence-constrained procedure.^11^ The candidate set is defined in eq 54

54 $$P_{cand}^{\left(t\right)}=\left\{\left\{i,j\right\}|d_{ij}^{\left(t\right)}<\eta_{cov}\left(r_{i}^{cov}+r_{j}^{cov}\right)\right\},,\eta_{cov}=1.3$$

where *r*_*i*_^cov^ denotes the element-specific covalent radius (in Å) of atom *i* taken from the Mendeleev database.^18^ The known total charge of the system *Q*_total_ is used in the valence-constrained assignment procedure. A per-snapshot time limit of τ = 5 s is imposed.

The procedure is applied independently to all 180 held-out snapshots per system. Connectivity and covalent states are read from the resulting RDKit bond objects, while formal charges and unpaired electron counts are read from the corresponding atom attributes. Therefore, the RDKit baseline is evaluated using the same bond, conditional bond order, formal charge, unpaired electron, valid, and Ave. problem definitions: CoTAR. A timeout or incomplete assignment sets all unresolved outputs to unknown and remains in every applicable denominator; no temporal smoothing is applied to RDKit.

#### Downstream Validation

To assess downstream utility, the CoTAR-predicted bond graph, bond orders, and formal charges are connected to OpenFF 2.0.0 and simulated in OpenMM.^22,23^ The main analysis uses the full pipeline after HMM smoothing; an ablation compares frame 0 predictions before and after smoothing. We evaluated simulation feasibility and consistency with the reference-topology density using the same partial-charge procedure for both topologies.

For 110 systems that have completed both simulations, longer runs are used to compare the density, diffusion coefficients, and radial distribution functions. Detailed settings are provided in Supporting Information.

## Results and Discussion

We first estimated the computational cost and then the predictive accuracy and contribution of individual components. Subsequently, we assessed external system transfer and the usability of the reconstructed topologies in downstream classical MD. Unless otherwise noted, CoTAR denotes the full pipeline with the vdW prior, chemical constraints, and HMM smoothing enabled. In Table 1, CoTAR denotes the pretrained model for classical-MD evaluation and the fine-tuned model for uMLIP evaluation.

### Inference Speed

Figure 2 shows inference time per snapshot as a function of the atom count. The vdW heuristic was the fastest method, CoTAR scaled smoothly to the largest tested system (100,005 atoms), and RDKit showed the steepest increase in cost. The steeper RDKit scaling is consistent with the increasing cost of rule-based bond order assignment on large, dense candidate graphs. Million-atom systems were not evaluated and would likely require graph partitioning or chunked inference. Hardware specifications, timing methodology, and representative wall-clock times are provided in Supporting Information.

**Figure 2:**
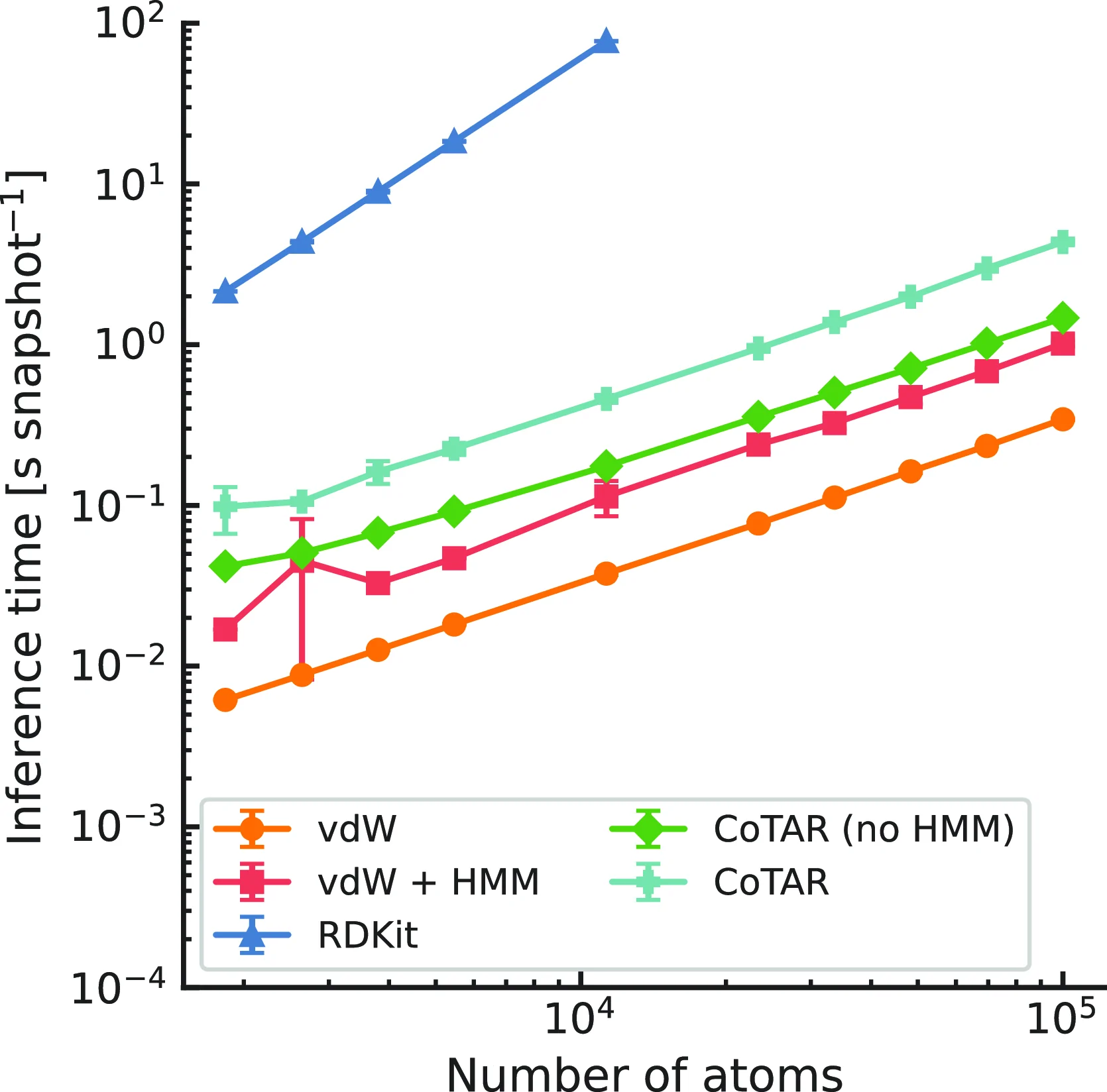
Inference time per snapshot in a separate liquid-toluene scaling test. All methods used the same configuration; hardware and timing details are given in Supporting Information.

### Basic Predictive Accuracy

Table 1 presents the category-resolved and category-macro results for the classical MD and uMLIP benchmarks. CoTAR and RDKit reconstructed connectivity, bond order, formal charge, and unpaired electrons, whereas the vdW baselines predicted only connectivity.

For classical MD trajectories, CoTAR achieved a category-macro conditional bond-order F1 of 0.999 and valid snapshots of 94.0%. RADICAL systems exhibited the lowest valid snapshot rate in this benchmark (76.5%).

For uMLIP trajectories, CoTAR achieved category-macro-bond and conditional-bond-order F1 scores of 0.9924 and 0.9984, respectively, with 82.1% valid snapshots. ION systems were the most difficult category, with 61.0% valid snapshots. RDKit achieved 58.2% valid snapshots, while the vdW and vdW + HMM baselines achieved 36.8% and 37.6%, respectively. This shows that high connectivity accuracy alone is insufficient for a chemically complete reconstruction.

The near-saturated pair-level F1 scores together with a lower snapshot validity rate indicate that a small number of atom-state or charge errors can invalidate an otherwise accurate reconstruction. Because each benchmark system contributed only 20 labeled frames to the pooled joint fine-tuning set, the main result demonstrates low-label adaptation to uMLIP trajectories within the represented systems rather than zero-shot generalization; unseen system transfer is assessed separately below.

### Ablation Study

Table 2 presents the results of a controlled ablation using 450 fine-tuning and 50 evaluation snapshots per compound, with each variant trained for 5 epochs. Because this protocol differs from the main transfer evaluation, the rows compare the component contributions rather than reproduce Table 1B.

HMM smoothing provided a modest but consistent final refinement. Removing HMM decreased the valid-snapshot rate from 98.9% to 97.7%, increased the average number of problems from 0.02 to 0.04, and reduced BO and FC F1 from 0.9970 and 0.9966 to 0.9945 and 0.9911, respectively.

A larger practical effect arose from the chemical constraints. Without HMM, the model retained chemical constraints but omitted the vdW prior, achieving 97.4% valid snapshots and 0.24 average problems. By contrast, configurations without chemical constraints demonstrated 3.07–6.23 problems per snapshot and an FC F1 of 0.9899.

The vdW prior exerted a smaller context-dependent effect. In the presence of chemical constraints, it improved the valid snapshot rate from 97.4% to 97.7% and reduced the average number of problems from 0.24 to 0.04, with the local F1 values remaining almost unchanged. These results identify chemical constraints as the main contributor to chemically usable assignments, with HMM acting as a cleanup step and the vdW prior being the most useful in combination with constraints.

### External-System Zero-Shot Evaluation

Table 3 presents the results for six complete external systems evaluated on 200 frames uniformly subsampled from each 5001-frame production trajectory and processed in chronological order without target-system training, fine-tuning, or parameter tuning. The model and all of the inference settings were fixed before evaluation. Related elements, constituent species, and local motifs could occur in the benchmark data; therefore, this experiment assessed transfer to unseen systems rather than completely disjoint chemistries.

**Table 3:** Zero-Shot Results after HMM Decoding of 200 Frames Uniformly Subsampled from Each 5001-Frame Production Trajectory and Processed in Chronological Order^a^

system	category	bond	BO	FC	UE	valid (%)	avg. prob	Q agree (%)
glyme	pure liquid	1.0000	1.0000	1.0000	1.0000	100.0	0.00	100.0
acetonitrile	pure liquid	1.0000	0.8000	1.0000	1.0000	100.0	0.00	100.0
urea/water	solution	1.0000	1.0000	1.0000	1.0000	100.0	0.00	100.0
TMACl/water	ion	1.0000	1.0000	0.9839	1.0000	0.0	18.00	80.5
ChCl/EG	ion	1.0000	1.0000	0.9292	1.0000	0.0	72.00	100.0
TEMPONE/water	radical	1.0000	1.0000	1.0000	1.0000	100.0	0.00	100.0

a Six complete external systems are excluded from all training, fine-tuning, and target-system tuning. BO, FC, UE, and *Q* agree denote conditional bond-order, formal-charge, unpaired-electron F1, and total-charge agreement, respectively.

After HMM decoding, the four tested nonionic systems yielded 100% of chemically valid snapshots. Acetonitrile retained a lower conditional bond-order F1 of 0.8000, showing that snapshot-level chemical validity does not imply the exact recovery of every reference bond order. By contrast, both ionic mixtures yielded 0% valid snapshots because of persistent formal charge assignment errors. HMM smoothing removed intermittent errors in acetonitrile and TEMPONE/water but did not eliminate systematic ionic errors.

Ionic failures are consistent with the limited diversity of the ION subset, which contains only NaCl, CaCl_2_, and NaOH solutions, while the external systems contain organic cations with different charge localization environments. Although the successful TEMPONE/water result is encouraging, the RADICAL subset contains only five species and includes the related TEMPO motif. A broader zero-shot transfer to ionic and radical chemistries, therefore, remains to be established.

### Reconnection to Classical MD

We next tested whether the CoTAR topologies could be propagated using a classical force field. Of the 128 uMLIP systems, 110 (85.9%) completed the downstream validation. HMM smoothing increased the success rate from 83.6% to 85.9%. RADICAL systems were the most difficult in the classical-MD snapshot reconstruction benchmark, while ION systems were the most difficult at the downstream reconnection stage; because these results are related to different data sets, stages, and metrics, they are not contradictory.

For the successful systems, simulations using the reconstructed topologies reproduced the reference densities, diffusion coefficients, and radial distribution functions (Figure 3).

**Figure 3:**
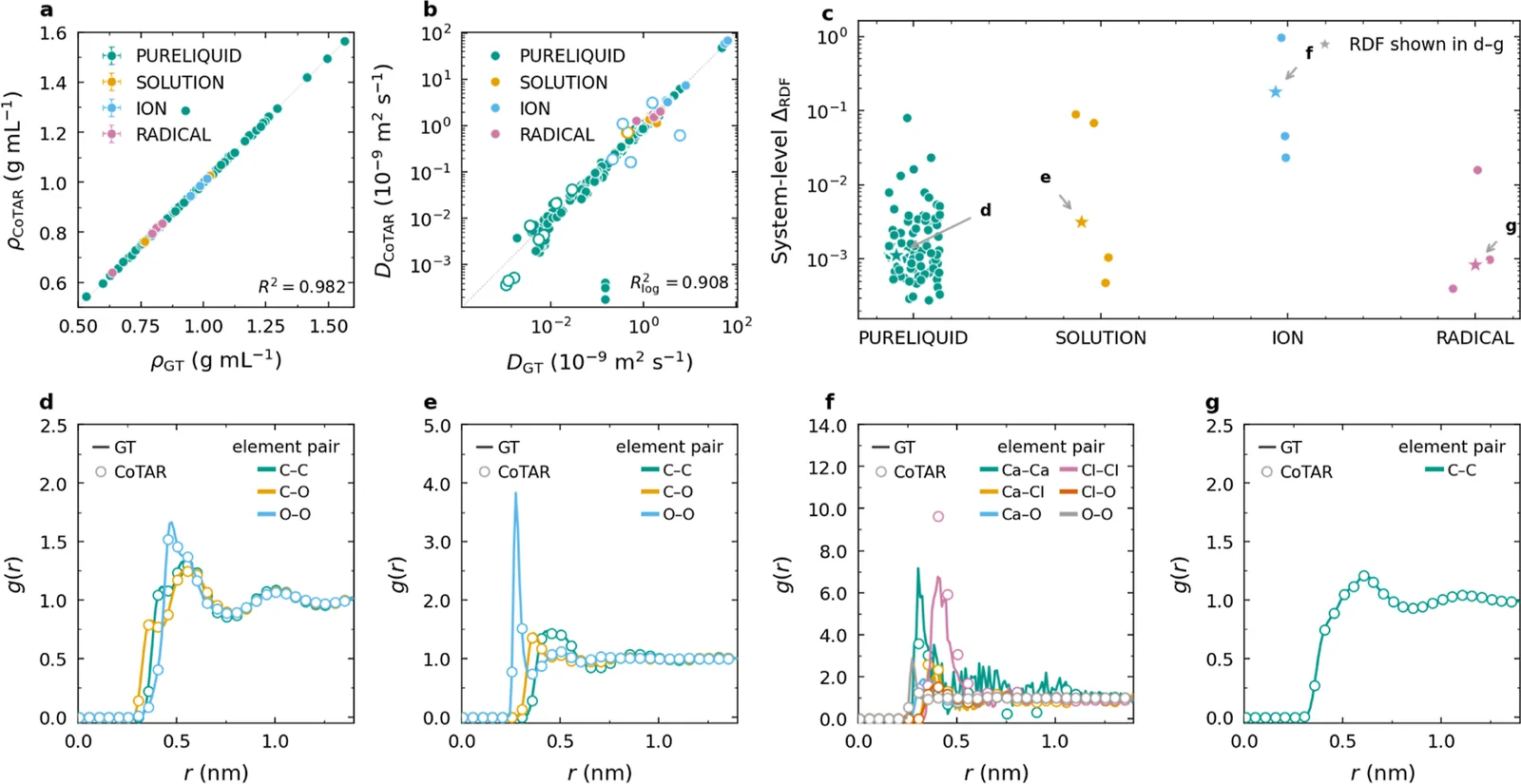
Downstream classical-MD comparison for reference (GT) and CoTAR topologies: (a) density, (b) diffusion coefficients, (c) RDF discrepancy, and (d–g) representative RDFs. Open symbols in (b) denote unstable estimates; dotted lines indicate identity and ±20%.

The density comparison gave *r* ^2^ = 0.982, and the stable diffusion estimates gave *r*_log_^2^ = 0.908. The RDF differences were generally small, with greater variability observed for a smaller ION subset.

Agreement in these observables indicates that for systems that can be reconnected successfully, the reconstructed topologies preserve both bulk packing and molecular mobility under the same force-field workflow. The failure concentration in the ION systems shows that high bond-level accuracy alone is not sufficient when charge assignment or subsequent force field typing is incorrect, while the modest improvement achieved with HMM smoothing indicates that temporal refinement removes intermittent defects but not systematic chemical errors. The downstream tests therefore support CoTAR as a practical bridge to classical MD for successfully reconstructed, topology-preserving systems, but they do not replace the validation of the force field itself.

### Interpretation, Scope, and Limitations

The present method is limited to nonreactive, topology-preserving condensed-phase systems. External evaluation involves only six systems, and the ION and RADICAL subsets contain relatively few chemistries; therefore, the zero-shot results should not be generalized to a broad ionic or open-shell chemical space. Atomic coordinates together with the reconstructed bond orders, formal charges, and unpaired-electron states provide a discrete molecular description required for the present analyses and force-field reconnection but not a complete electronic description. will require additional prediction targets; furthermore, vector or tensor multipoles will require an equivariant output representation.

The main uMLIP benchmark uses labeled frames from every evaluation system during joint fine-tuning and therefore measures low-label in-domain adaptation rather than unseen system generalization. Moreover, 109 of the 128 benchmark systems are PURELIQUID; category-macro reporting prevents this class from dominating the summary statistic but does not replace broader ionic and radical coverage.

CoTAR has no explicit molecular-weight cutoff. Its accuracy was evaluated for systems containing approximately 1000–3300 atoms, and computational scaling was measured up to 100,005 atoms. The benchmarks were generated at approximately 300 K and 1 atm; therefore, its performance outside these conditions has not been estimated. First-order phase transitions such as melting or freezing were not excluded in principle if covalent topologies remain unchanged, but trajectories crossing such transitions were not evaluated in this study. More generally, topology-preserving conformational changes are within the scope, while processes involving bond formation or cleavage are outside the present formulation.

The downstream tests compare the reference and reconstructed topologies within a common force field workflow and do not assess the absolute accuracy of the force field.

## Conclusions

We developed CoTAR, a hybrid GNN–HMM framework for reconstructing the molecular topology, formal charges, and unpaired electrons from condensed-phase trajectories. Across more than 100 different nonreactive systems, CoTAR outperformed distance-based and rule-based baselines and provided chemically valid reconstructions across all four benchmark categories. Zero-shot tests showed strong transfer for the tested nonionic systems, while ionic mixtures remained limited by formal-charge assignment.

The reconstructed topologies supported classical MD and reproduced reference densities, diffusion coefficients, and radial distribution functions. Chemical constraints were primarily responsible for chemically usable assignments, while HMM smoothing provided final temporal refinement. The vdW prior offered a smaller, context-dependent benefit and was most useful when used together with constraints. The contrast between the adapted benchmark and external zero-shot tests indicates that modest per-system labeling can transfer the pretrained representation to uMLIP trajectories within represented chemical environments, while unfamiliar ionic charge localization remains the main obstacle to broader generalization. The current scope is limited to topology-preserving systems and the represented chemical environments; broader ionic and radical coverage and extension to bond-changing processes remain important directions for future studies.

## Supplementary Material





## Data Availability

An evaluation release of the CoTAR software is publicly available at https://github.com/pfnet-research/cotar. Machine-readable numerical data underlying Figures 2 and 3 and Tables 1–3 are provided as Supporting Information. These data enable reproduction of the plots and summary statistics reported in this study.

## Notes

Machine-readable numerical data underlying Figures 2 and 3 and Tables 1–3 (ZIP)
System inventory and dataset composition; benchmark and zero-shot results; computational-scaling details; and downstream classical-MD protocols for density, diffusion coefficients, and radial distribution functions (PDF)
