# Supplementary material for: CoTAR: Topology and Atomic State Reconstruction in Condensed Phases
Source: J Chem Inf Model. 2026 Sep 18;66(19):12507–17. doi: 10.1021/acs.jcim.6c02184 (PMC13663487; doi:10.1021/acs.jcim.6c02184)
Supplement: Supplementary file 1 [file ci6c02184_si_002.pdf]

Supporting Information: Supporting Information for "CoTAR: Topology and Atomic State Reconstruction in Condensed Phases"

Hodaka Mori,* Yu Miyazaki, and Takechika Kikkawa

Preferred Networks, Inc., Tokyo 100-0004, Japan

E-mail: hodakamori@preferred.jp

Complete system list and dataset composition

Table S1 lists the 128 benchmark systems and 6 complete external systems used for zero-shot evaluation. The benchmark identifiers are grouped by category and arranged in two columns while preserving the internal dataset naming convention. The external systems are listed separately and were excluded in their entirety from all pretraining, fine-tuning, model selection, and target-system tuning.

Table S1: Benchmark systems and complete external zero-shot systems grouped by category.

**PURELIQUID** (109 systems)	
`1_1_1_trichloro_2_2_2_trifluoroethane`	`1_1_1_trichloroethane`
`1_1_2_trichloro_1_2_2_trifluoro_ethane`	`1_1_2_trichloroethylene`
`1_1_dichloroethane`	`1_1_dichloroethene`

Continued on next page

Table S1 (continued)

1_2_dichlorobenzene	1_2_dichloroethane
1_3_dioxolan_2_one	1_4_diethylbenzene
1_4_dioxane	1_methyl_2_pyrrolidone
1_nitroethane	2_2_2_hydroxyethoxyethoxyethanol
2_2_4_trimethylpentane	2_2_butoxyethoxyethanol
2_2_ethoxyethoxyethanol	2_2_hydroxyethoxyethanol
2_2_methoxyethoxyethanol	2_2_methoxypropoxypropan_1_ol
2_6_dimethylheptan_4_one	2_aminoethanol
2_butoxyethanol	2_ethoxyethanol
2_hydroxybenzoicacidmethylester	2_methylpropan_1_ol
2_methylpropionicacidisobutylester	2_nitropropane
3_5_5_trimethylcyclohex_2_en_1_one	4_hydroxy_4_methyl_pentan_2_one
4_methyl_1_3_dioxol_2_one	4_methylpentan_2_one
5_methylhexan_2_one	aceticacid
aceticacid2_ethoxyethylester	aceticacidbutylester
aceticacidethylester	aceticacidisoamylester
aceticacidisobutylester	aceticacidmethylester
acetone	acetophenone
aminopropyltrimethoxysilane	aniline
benzene	benzoicacid
butan_1_ol	butan_2_ol
butan_2_one	carbondisulfide
carbonicaciddiethylester	carbontetrachloride
chlorobenzene	chloroform
chloromethane	cyclohexane
cyclohexanol	cyclohexanone
cyclohexylamine	decan_1_ol
dichloromethane	dodecane
ethane_1_2_diamine	ethanethiol

Continued on next page

Table S1 (continued)

ethanol	ethoxyethane
ethylbenzene	ethyleneglycol
formamide	formicacid
formicacidethylester	glycerol
m_cresol	methanol
methylcyclohexane	methylsulfinylmethane
morpholine	n_butane
n_heptane	n_hexane
n_n_dimethylformamide	n_octane
n_pentane	naphthalene
nitrobenzene	nitromethane
o_xylene	octamethylcyclotetrasiloxane
oleicacid	pentan_3_one
pentyl_acetate	phenol
phenylmethanol	phenylmethoxymethylbenzene
phenyltriethoxysilane	prop_2_en_1_ol
propan_1_ol	propan_2_ol
propane_1_2_diol	pyridine
quinoline	resorcinol
stearicacid	styrene
sulfuricaciddiethylester	tetraethoxysilane
tetrahydrofuran	toluene
water	
**SOLUTION** (5 systems)	
water_alanine_dipeptide	water_ethanol_0_100
water_ethanol_100_0	water_ethanol_50_50
water_glycine	
**ION** (9 systems)	

Continued on next page

Table S1 (continued)

cacl2_0.1M	cacl2_1M
cacl2_10M	nacl_0.1M
nacl_1M	nacl_10M
naoh_0.1M	naoh_1M
naoh_10M	
**RADICAL** (5 systems)	
allyl_radical	benzyl_radical
phenoxyl_radical	tempo
tert_butyl_radical	
**EXTERNAL ZERO-SHOT** (6 systems; excluded from all training and target-system tuning)	
Glyme: 188 molecules (3,008 atoms)	Acetonitrile: 500 molecules (3,000 atoms)
Urea/water: 18 urea + 1,000 water (3,144 atoms)	TMACl/water: 18 TMA^+ + 18 Cl^- + 1,000 water (3,324 atoms)
ChCl/EG: 72 $choline^+$ + 72 Cl^- + 144 EG (3,024 atoms)	TEMPONE/water: 1 TEMPONE + 1,000 water (3,028 atoms)

The six complete external systems were absent from the benchmark inventory and were not used at any stage of training or target-system tuning. Some constituent species and local motifs, including water, chloride, ethylene glycol, and the related TEMPO motif, were represented in the benchmark systems. The zero-shot experiment therefore evaluated transfer to unseen complete systems rather than extrapolated to fully disjoint chemical elements or motifs.

Table S2 summarizes the benchmark system counts and exact fine-tuning and held-out frame totals used in main evaluations.

Classical-MD trajectories from all 128 benchmark systems were used for pretraining, and all 128 systems were included in classical-MD evaluation presented in Table 1A. The same 128 systems were also included in the main uMLIP benchmark.

For each uMLIP benchmark system, a fixed manifest contained exactly 200 frames. A total of 20 labeled frames from each of the 128 systems were pooled into a 2,560-frame

Table S2: System counts for main classical-MD evaluation and system/frame counts for main uMLIP evaluation in Table 1.

	Inventory	Classical-MD	uMLIP evaluation		
Category	systems	eval. systems	systems	fine-tune frames	held-out frames
PURELIQUID	109	109	109	2,180	19,620
SOLUTION	5	5	5	100	900
ION	9	9	9	180	1,620
RADICAL	5	5	5	100	900
Total	128	128	128	2,560	23,040

training set, which was then used to jointly fine-tune a single pretrained CoTAR model; the remaining 180 disjoint frames per system formed the common held-out evaluation set for CoTAR, vdW, vdW+HMM, and RDKit. HMM-based methods processed the 180 held-out frames in the chronological order, while RDKit was applied independently with a 5 s limit per snapshot. No frame was removed after observing a prediction, failure, or timeout, and failures and timeouts remained in all applicable denominators.

The ablation study (results presented in Table 2) followed a separate protocol with exactly 450 fine-tuning frames and 50 disjoint evaluation frames per compound. These counts differed from the 20/180 split used in the main uMLIP benchmark; Table 2 is therefore intended for a controlled comparison of model variants rather than a direct comparison with Table 1B.

The benchmark inventory was strongly imbalanced: 109 of 128 systems are PURELIQUID, compared with 5 SOLUTION, 9 ION, and 5 RADICAL systems. Additional external tests cannot remove this imbalance. Instead, the primary aggregate is an unweighted macro-average over the four categories, and the category-resolved results are reported such that smaller ION and RADICAL subsets remain visible. Although the six external systems present challenging ionic and open-shell cases, they are not presented as a balanced replacement benchmark.

Metric aggregation and uMLIP summary

For the uMLIP comparison, classification confusion matrices were pooled within each category before F1 calculation, and the Valid and average-problem values were pooled over snapshots within each category. Because the system counts are 109/5/9/5 across PURELIQUID/SOLUTION/ION/RADICAL, respectively, the primary category-macro value was the unweighted mean of the four category results; therefore, each category contributed equally despite unequal system counts. Conditional BO F1 included only pairs that were covalent in both reference and prediction; missed bonds were penalized by Bond F1 and snapshot validity. Failures and timeouts remained in all applicable denominators. RDKit connectivity, bond order, formal charge, and unpaired-electron outputs were evaluated using the same metric implementation as CoTAR. Table S4 additionally shows the effects of category-macro, system-macro, and fully pooled aggregation on the classical-MD summary.

Table S3 summarizes the category-macro uMLIP results discussed in the main text.

Table S3: Category-macro uMLIP results. BO denotes conditional bond-order F1; vdW baselines predict only connectivity.

Metric	Method	Category macro
Bond F1	vdW	0.9814
	vdW + HMM	0.9815
	RDKit	0.6561
	CoTAR	**0.9924**
Conditional BO F1	RDKit	0.6383
	CoTAR	**0.9984**
Formal-charge F1	RDKit	0.6513
	CoTAR	**0.9969**
Unpaired-electron F1	RDKit	0.6520
	CoTAR	**0.9996**
Valid snapshots (%)	vdW	36.8
	vdW + HMM	37.6
	RDKit	58.2
	CoTAR	**82.1**
Avg. problems/snapshot	vdW	88.12
	vdW + HMM	87.49
	RDKit	3,414.88
	CoTAR	**0.82**

RDKit achieved 58.2% category-macro validity, but 0% for ION, demonstrating 3,414.88 problems per snapshot on average. CoTAR led all six category-macro metrics and exhibited nonzero validity in every category. Confidence intervals in Table S4 used 10,000 system-level bootstrap replicates (seed 2026), with stratified resampling used for category-macro estimates.

Table S4: Classical-MD aggregation sensitivity; parentheses give 95% system-bootstrap confidence intervals.

Aggregation	BO F1	Valid (%)	Avg. problems
Category macro	0.9986 (0.9971–0.9997)	94.0 (89.7–97.8)	0.170 (0.062–0.301)
System macro	0.9956 (0.9901–0.9997)	98.7 (97.3–99.7)	0.035 (0.007–0.072)
Fully pooled	0.9969 (0.9929–0.9998)	99.4 (98.7–99.9)	0.017 (0.003–0.037)

Additional zero-shot results

Zero-shot protocol

For each external system, 200 frames were uniformly subsampled from the 5,001-frame production trajectory and processed chronologically. Except that target-system fine-tuning was omitted and all 200 frames were evaluated, all other data-generation and evaluation conditions matched the main uMLIP benchmark. Each complete system was excluded from pretraining, fine-tuning, model selection, threshold selection, and HMM-parameter selection; the model and all settings were fixed using only the 128 benchmark systems before evaluation.

The protocol evaluated transfer to unseen complete systems, not fully chemistry-disjoint extrapolation, because some constituent species and local motifs occurred in the benchmark inventory. Valid measured chemical consistency, not exact reference-topology recovery, and should be interpreted with Bond and conditional BO F1. For example, acetonitrile reached 100% Valid after HMM decoding, while its conditional BO F1 was 0.8000. Tables S5 and S6 present the framewise results before HMM smoothing and the corresponding changes after HMM decoding.

Table S5: Frame-wise zero-shot results before HMM smoothing for 200 frames uniformly subsampled from each 5,001-frame production trajectory and processed in the chronological order. BO denotes conditional bond-order F1.

System	Category	BO	FC	UE	Valid (%)	Avg. prob.
Glyme	PURELIQUID	1.0000	1.0000	1.0000	100.0	0.00
Acetonitrile	PURELIQUID	0.7991	1.0000	1.0000	91.0	0.09
Urea/water	SOLUTION	0.9999	1.0000	1.0000	100.0	0.00
TMACl/water	ION	1.0000	0.9838	1.0000	0.0	18.00
ChCl/EG	ION	1.0000	0.9292	1.0000	0.0	72.00
TEMPONE/water	RADICAL	1.0000	1.0000	1.0000	98.5	0.02

Table S6: Effect of HMM decoding on zero-shot validity and problem counts over 200 frames uniformly subsampled from each 5,001-frame production trajectory and processed in the chronological order.

System	Valid no HMM (%)	Valid HMM (%)	Problems no HMM	Problems HMM	Q agree (%)
Glyme	100.0	100.0	0.00	0.00	100.0
Acetonitrile	91.0	100.0	0.09	0.00	100.0
Urea/water	100.0	100.0	0.00	0.00	100.0
TMACl/water	0.0	0.0	18.00	18.00	80.5
ChCl/EG	0.0	0.0	72.00	72.00	100.0
TEMPONE/water	98.5	100.0	0.02	0.00	100.0

Computational scaling conditions

The scaling benchmark used periodic liquid toluene cells of increasing size. The job was allocated to one NVIDIA A100 GPU with 80 GB of device memory, eight CPU cores on an AMD EPYC 7713 processor, and a 64 GiB host-memory limit. CoTAR inference ran on the GPU, while the vdW and RDKit baselines and HMM postprocessing ran on the CPU. Each value is an average of three wall-clock measurements; the runtime of a five-frame sequence was divided by five for HMM-enabled methods.

The representative wall-clock values for the scaling benchmark are listed in Table S7.

Table S7: Liquid-toluene wall-clock times; RDKit was not run at 100,005 atoms.

	Time (s snapshot^{-1})			
Atoms	CoTAR no HMM	CoTAR with HMM	RDKit	Repeats
1,830	0.0418	0.0983	2.145	3
11,295	0.1755	0.4602	77.334	3
100,005	1.468	4.354	–	3

Simulation details for downstream classical-MD validation

Unless otherwise noted, the classical-MD simulations used for downstream validation were performed with OpenMM 8.1.1. This section summarizes system construction, force-field

assignment, and the simulation protocol used to connect the reference (GT) and CoTAR-predicted topologies to a classical force field.

Classical-MD setup

The OpenFF 2.0.0 force field was used for estimating bonded and nonbonded interactions. For chemical environments not directly covered by the standard parameter set, auxiliary bonded parameters were generated by fitting to the potential-energy surfaces obtained from PFP calculations.[S1] Partial charges were assigned via Bader charge analysis using PFP (v6.0.0) and uniformly scaled by a factor of 0.6.

Electrostatic interactions were treated using the particle-mesh Ewald method. A real-space cutoff of 0.9 nm was used, with a switching function applied from 0.8 nm. Additionally, van der Waals interactions were truncated at 0.9 nm, and long-range dispersion corrections were applied to the energy and pressure.

The feasibility of simulations was determined in three stages: NVT equilibration

for 10 ps, NPT equilibration

for 150 ps, and NPT production

for 250 ps. The temperature was set to 300 K throughout, and the pressure was set to 1 atm during the NPT stages. Temperature control used a Langevin thermostat with a coupling constant of 1.0 ps^{-1}, and pressure control used a Monte Carlo barostat. A time step of 0.5 fs was used, and all bonds involving hydrogen atoms were constrained using the RATTLE algorithm. The same protocol was applied to the GT and CoTAR-predicted topologies.

Of the 128 systems, 110 completed both simulations: 97 PURELIQUID, 5 SOLUTION, 4 ION, and 4 RADICAL systems. These systems were then simulated for 100 ps of NVT equilibration, 1.5 ns of NPT equilibration, and 2.5 ns of NPT production using the same force-field and integration settings. The production trajectories were used for the density, diffusion-coefficient, and radial-distribution-function analyses described below.

Density

The instantaneous density was calculated every 0.05 ps using Equation S1:

$$\rho(t) = \frac{M}{V(t)}, \tag{S1}$$

where M is the total system mass and $V(t)$ is the instantaneous periodic-cell volume. The values obtained in the first 1.25 ns of production were discarded, and the reported density was the arithmetic mean of the remaining 25,001 samples over 1.25–2.50 ns. Error bars in Figure 3a denote one population standard deviation. The displayed r^2 was the squared Pearson correlation among the paired mean densities over the 110 systems; no outlier removal was applied.

Diffusion coefficients

The production coordinates were saved every 0.5 ps and unwrapped across PBCs. For element α, the diffusion coefficient was estimated from the displacement of atoms of that element using Equation S2

$$D_\alpha = \frac{1}{2d}\frac{d}{d\tau}\left\langle \|\mathbf{r}_i(t_0+\tau) - \mathbf{r}_i(t_0)\|^2 \right\rangle_{i\in\alpha,t_0}, \qquad d = 3, \tag{S2}$$

where the average was calculated over all atoms of element α and all available time origins t_0. The slope was obtained by linear regression over lag times of 500–1500 ps. A paired estimate was classified as reliable only when both GT and CoTAR results contained at least 10 atoms of the element; the primary estimate was finite and positive; the primary fit had $R^2 \geq 0.98$; estimates from the 250–1000, 500–1250, 500–1500, and 750–1500 ps windows were finite and positive; and their range divided by the primary estimate did not exceed 0.30. Among 329 paired estimates, 311 satisfied these criteria. The remaining 18 are shown as open symbols in Figure 3b and were excluded from the displayed $r^2_{\log}$, defined as the

squared Pearson correlation between $\log_{10} D_{\mathrm{GT}}$ and $\log_{10} D_{\mathrm{CoTAR}}$ for the reliable estimates.

Radial distribution functions

Hydrogen atoms were excluded, and the remaining atoms were grouped by element. Molecular membership was defined independently for the GT and CoTAR topologies from the connected components of the corresponding bond graph; all same-molecule pairs were excluded. Distances were evaluated based on the minimum-image convention using the instantaneous periodic box. RDFs were calculated on a common grid of 140 bins over $0 \leq r < 1.4$ nm with a bin width of 0.01 nm and normalized using the instantaneous cell volume.

For element pair p in system s, the curve discrepancy was defined by Equation S3

$$\Delta_{s,p} = \frac{1}{K} \sum_{k=1}^{K} \left| g_{s,p}^{\mathrm{CoTAR}}(r_k) - g_{s,p}^{\mathrm{GT}}(r_k) \right|, \qquad K = 140. \tag{S3}$$

The system-level discrepancy was the unweighted mean over the matched element pairs in Equation S4

$$\Delta_{\mathrm{RDF},s} = \frac{1}{P_s} \sum_{p=1}^{P_s} \Delta_{s,p}. \tag{S4}$$

In Figure 3c, black horizontal lines denote category medians and gray bars denote interquartile ranges. The representative system for each category was the one whose $\Delta_{\mathrm{RDF},s}$ was closest to the category median, with the lexicographic system ID used to break ties.

References

(S1) Matlantis, software as a service style material discovery tool. `https://matlantis.com/`.
